# Proteomics, Bioinformatics and Structure-Function Antigen Mining For Gonorrhea Vaccines

**DOI:** 10.3389/fimmu.2018.02793

**Published:** 2018-12-04

**Authors:** Benjamin I. Baarda, Fabian G. Martinez, Aleksandra E. Sikora

**Affiliations:** ^1^Department of Pharmaceutical Sciences, College of Pharmacy, Oregon State University, Corvallis, OR, United States; ^2^Vaccine and Gene Therapy Institute, Oregon Health and Science University, Beaverton, OR, United States

**Keywords:** *Neisseria gonorrhoeae*, vaccine, proteomics, antigen, reverse vaccinology, bioinformatics, protein structure-function, membrane vesicles

## Abstract

Expanding efforts to develop preventive gonorrhea vaccines is critical because of the serious health consequences combined with the prevalence and the dire possibility of untreatable gonorrhea. Reverse vaccinology, which includes genome and proteome mining, has proven successful in the discovery of vaccine candidates against many pathogenic bacteria. Here, we describe proteomic applications including comprehensive, quantitative proteomic platforms and immunoproteomics coupled with broad-ranging bioinformatics that have been applied for antigen mining to develop gonorrhea vaccine(s). We further focus on outlining the vaccine candidate decision tree, describe the structure-function of novel proteome-derived antigens as well as ways to gain insights into their roles in the cell envelope, and underscore new lessons learned about the fascinating biology of *Neisseria gonorrhoeae*.

## Introduction

Worldwide, over 78 million people are estimated to acquire the sexually transmitted infection gonorrhea every year (1). Female reproductive health is disproportionately affected by this disease and half of infected women show no symptoms (2–4). Serious health consequences are associated with untreated or insufficiently treated gonorrhea, including pelvic inflammatory disease, inflammation of the fallopian tubes, pre-term delivery, miscarriage, or ectopic pregnancy (5–7). Additionally, infants born vaginally to infected mothers are exposed to the disease in the birth canal and are thus at risk of developing a sight-threatening conjunctivitis (8). Although the health consequences to men are not as severe as for women and predominantly manifest as uncomplicated urethritis accompanied by a neutrophil-rich exudate (6, 9), gonorrhea can ascend to the epididymis or the testes and may require surgical removal of the infected site (10–12). Infertility can occur in both females and males without proper treatment (9, 13).

The bacterium responsible for gonorrhea, *Neisseria gonorrhoeae* (*Ng*), is a highly adaptable pathogen. Its natural competence and plastic genome have contributed to the extensive spread of antibiotic resistance. Through a number of horizontally acquired genes and chromosomal mutations, *Ng* has become resistant to every antibiotic used for its treatment (14–16). The Centers for Disease Control and Prevention (CDC) in the United States currently recommend a dual therapy of intramuscular ceftriaxone combined with oral azithromycin as a first-line treatment for uncomplicated gonorrhea (17, 18). However, the first isolates resistant to this combination therapy have begun to emerge (19). Three new therapeutics for gonorrhea treatment are being evaluated in clinical trials (20), but considering the speed with which the gonococcus develops antibiotic resistance (15), new drugs will not provide a long-term solution. The development and introduction of a protective vaccine against gonorrhea should therefore be prioritized to limit its spread.

Thus far, only two gonorrhea vaccines, using either killed whole organisms or purified pilin protein, have progressed to clinical trials. Despite robust antibody responses in both trials, neither vaccine provided protection against acquiring the disease after immunization (21–24). These failures are likely due to a number of factors. Pilin proteins undergo extensive antigenic variation through frequent recombination with transcriptionally silent *pilS* gene cassettes (25–28). Experimental infections have demonstrated that multiple pilin variants are isolated from a single individual, and that these variants are antigenically distinct from the inoculating parent strain (29–31). Further, pilin proteins are subjected to phase variation, where protein expression transitions between “on” and “off” states through slipped-strand repair of upstream repeat regions (32). Antigenic and phase variation of pilin during infection likely contributed to the failure of both vaccine trials. Another factor that may have led to the whole cell vaccine's inability to protect from infection is the presence of the reduction modifiable protein (Rmp; also known as protein III) in the vaccine. Localized to the outer membrane, Rmp is highly conserved and immunogenic, yet antibodies induced by this antigen actively prevent assembly of the complement membrane attack complex in immune serum (33, 34). These challenges illustrated the necessity for new approaches in gonorrhea vaccine development.

In the intervening years, vaccine progress has been slow. One of the difficulties is that *Ng* infection rarely, if ever, leads to an adaptive immune response (35–38). For this reason, mechanisms of protection against gonorrhea are unknown (24), which makes the evaluation of the potential efficacy of vaccine candidates prior to expensive immunization studies challenging. The serum bactericidal activity of antibodies generated during an immune response strongly predicts protection for vaccines against *N. meningitidis* [*Nm*; (39, 40)], a frequent causative agent of meningitis, so the ability of gonococcal antigens to elicit bactericidal antibodies is currently used as a surrogate mechanism of protection (41). Based on this criterion, 14 *Ng* antigens with functions in colonization and invasion, nutrient acquisition, and immune evasion have been proposed for inclusion in a gonorrhea vaccine [reviewed in (41)]. Immunization with each of the candidate proteins, cyclic loop peptides, or lipooligosaccharide epitope mimics elicited bactericidal antibodies, although studies for seven of the antigens were performed only in *Nm* (41).

Despite the difficulties in developing a vaccine against gonorrhea, several recent advances suggest that a protective vaccine is now within reach. The first was the development of a female mouse model of lower genital tract infection, in which mice are treated with 17-β estradiol and a cocktail of antibiotics to increase susceptibility to *Ng* and to reduce the overgrowth of vaginal commensal bacteria, respectively (42). This model has enabled the study of the immune response to gonococcal infection in a whole organism for which extensive genetic and immunological tools are available (24, 43, 44). A series of elegant studies, combining information gathered from experimental murine infections and tissue culture experiments, demonstrated *Ng* actively suppresses the generation of a productive adaptive immune response. Both mouse splenic mononuclear cells and human dendritic cells infected with *Ng* produced elevated levels of interleukin (IL)-6, tumor necrosis factor-α (TNF-α), IL-1β, and IL-23, a set of cytokines that promote terminal differentiation of T-cells toward T helper 17 (Th17) cells (45, 46). Production of IL-17 is a characteristic marker of a Th17 response and promotes neutrophil recruitment through the induction of granulocyte-colony stimulating factor and chemokines (45). In support of gonorrhea promoting Th17 differentiation during an active infection, elevated levels of IL-17 were discovered in female mice challenged with *Ng* (46). Gonococci are also able to divert T-cell differentiation away from an adaptive Th1/Th2 response by inducing the production of transforming growth factor (TGF)-β and IL-10 (47–49). Furthermore, *Ng* stimulates the differentiation of macrophages toward a regulatory phenotype and prevents macrophage antigen display. Through these immunosuppressive activities, the gonococcus is able to further inhibit the generation of a protective T-cell response (50, 51). The knowledge gained through these studies into the sophisticated methods *Ng* uses to promote its own survival and prevent triggering an adaptive immune response will enable a more informed strategy for vaccine development and help avoid the failures of the past.

In studies making use of the insights gathered from a better knowledge of the immune evasion strategies employed by the gonococcus, mice treated intravaginally with micro-encapsulated IL-12 and either infected with a common laboratory strain, FA1090, or immunized with membrane vesicles (MVs) collected from FA1090 were protected against subsequent infections up to 6 months after the initial treatment, even when challenged with heterologous strains (52, 53). IL-12 treatment promoted a Th1 response, as well as enhancing serum immunoglobulin A (IgA) and vaginal IgA and IgG levels (54).

Lessons can also be learned from the successful development of the licensed four-component *Nm* serogroup B vaccine, 4CMenB (BEXSERO; GlaxoSmithKline). This bacterium presented a daunting vaccination challenge for a number of years due to the polysaccharide capsule surrounding group B meningococci, which is structurally identical to the polysialic acid carbohydrate found on the surface of many human cells. Because of this similarity, immunization with the group B capsule is minimally immunogenic and/or may result in the generation of autoantibodies (55). To circumvent this problem, a subunit vaccine was developed by identifying conserved open reading frames in the whole genome sequence of *Nm* serogroup B, a strategy termed reverse vaccinology (55–59). Out of nearly 600 vaccine candidates identified with this strategy, 350 were successfully expressed and purified from *Escherichia coli*, 28 elicited bactericidal antibodies, and only three recombinant proteins—two of which are composed of a fusion of two proteins—were combined with MVs to formulate 4CMenB (59, 60). Finally, a retrospective study found that immunization with another *Nm* serogroup B vaccine, MeNZB, containing the same MVs as 4CMenB, was 31% effective at preventing gonorrhea (61, 62). The MeNZB vaccine is no longer available, but these seminal studies provide strong evidence that a protective gonorrhea vaccine is possible.

A comparison of the number of antigens evaluated for the serogroup B vaccine with the number currently being investigated for a gonorrhea vaccine illustrates how far gonorrhea research lags behind meningitis research and emphasizes that new strategies are necessary to increase the pool of vaccine candidates under consideration. An innovative way to address this gap was to perform reverse vaccinology antigen mining using subcellular fractionation coupled with high-throughput quantitative proteomics followed by bioinformatics (63, 64), which identified numerous stably expressed proteins and suggested that formulation of a subunit vaccine against gonorrhea would be successful. Both genome- and proteome-based reverse vaccinology approaches have become more prevalent since the technique was introduced (56). Candidate vaccine antigens have been identified through whole-genome screens of a number of medically important pathogens (65–69). As the availability of bacterial genome sequences has increased, more detailed analyses have become possible, including comparative genomics. One weakness of using whole genome sequences to search for vaccine candidate antigens is that the pathogens do not necessarily express the proteins discovered through this approach. Transcriptome analysis provides a way to circumvent this limitation but a low correlation between transcriptomic and proteomic data has been well established [reviewed in (70)]. For this reason, we have chosen to pursue proteomic-based reverse vaccinology, as proteomic studies reveal the biologically relevant population of proteins expressed during exposure to the conditions under examination (63, 64, 71). Proteomic approaches also have the potential to specifically identify surface-exposed proteins without the need for extensive bioinformatic predictions (72).

In this article, we provide an overview of proteomic and bioinformatic approaches that have been utilized for gonorrhea antigen mining. Our focus will also be on functional and structural characterization of proteome-derived antigens to determine their role in gonococcal pathogenesis and physiology as well as to inform the development of next generation vaccines based on structural vaccinology.

## Proteomic Technologies Applied for Gonorrhea Antigen Mining

**Figure 1:**
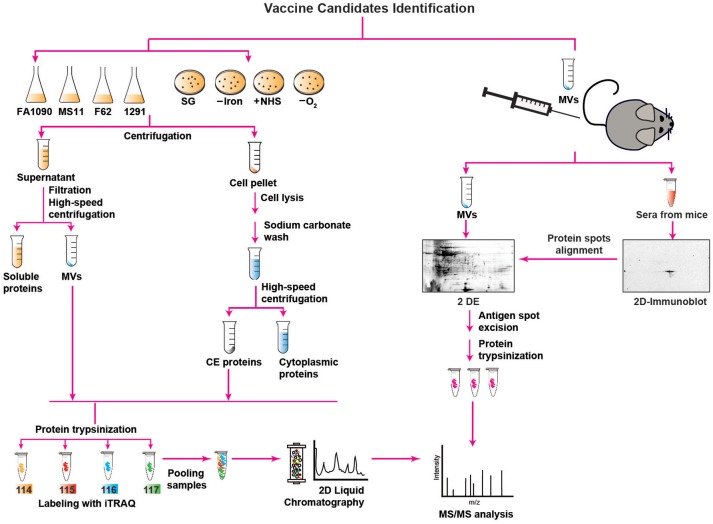
Outline of proteomics approaches used for mining of *Neisseria gonorrhoeae* vaccine antigens. Description is provided in the main text. SG, standard growth conditions; –Iron, iron deprivation; +NHS, addition of normal human serum; –O_2_, anaerobic growth conditions; MVs, membrane vesicles; CE, CE; 2 D, two-dimensional; 2 DE, two-dimensional electrophoresis; iTRAQ, isobaric tagging for absolute quantitation; MS/MS, mass spectrometry analysis.

Surface-localized proteins represent attractive vaccine candidates, as they are important foci for the immune system and play pivotal roles in bacterial physiology as well as host-pathogen interactions. Naturally elaborated MVs (NeMVs) and MVs extracted from cell evelopes (CE) by either lithium or deoxycholate treatment (LeOMVs or DeOMVs, respectively) contain surface-localized proteins, other outer membrane and periplasm-derived proteins, and commonly cytoplasmic proteins (73–79). In addition to proteins, MVs contain lipopolysaccharide and DNA of chromosomal, plasmid, or phage origin, as well as RNA (73, 75, 80). NeMVs are purified and concentrated from culture supernatants by separating intact cells from already-formed NeMVs (Figure 1). Le-MVS and De-MVs are extracted from bacterial cells with detergent, reducing the content of reactogenic LPS/LOS and also many lipoproteins (75, 81, 82). Including different types of MVs in vaccine formulations has led to some of the most effective vaccines against bacterial diseases (61, 81, 83). Remarkably, following a nationwide implementation of 4CMenB, a recent study showed >80% vaccine-mediated protection against current *Nm* B strains in the United Kingdom (84, 85).

For all these reasons, the identification of CE and MV antigens is a key objective of proteomics-driven vaccinology. It is however, a difficult task, because membrane proteins are commonly low abundant, hydrophobic, have a basic charge, and can be of high molecular weight. Therefore, comprehensive antigen mining of bacterial CE and MVs necessitates extensive subcellular fractionation procedures coupled with high-throughput quantitative proteomics and extensive bioinformatics (Figure 1). In addition, multidimensional protein identification technology combats these challenges by using a combination of two different kinds of liquid chromatography (2D-LC) that separates proteins prior to their identification, greatly diminishing the complexity of the sample at the peptide level and resulting in the identification of a superior number of proteins (75, 86).

Accordingly, to interrogate the *Ng* CE and MVs for new antigens, three independent proteomic technologies and experimental designs have been applied for the first time in the gonorrhea field (52, 63, 64). In the earliest proteomic mining, four common laboratory *Ng* strains (FA1090, MS11, F62, and 1291) were cultured under standard growth conditions in liquid medium to mid-logarithmic phase and subjected to fractionation, sodium carbonate extraction, and ultracentrifugation steps to isolate NeMVs and CE proteins [Figure 1; (63)]. Subsequently, these subproteomes were trypsinized and labeled with four different isobaric tags (114, 115, 116, and 117) targeting ε-amine group of lysine in peptides for relative and absolute quantitation (iTRAQ). After labeling, the samples were pooled and the peptide mixture was subjected to fractionation by 2D-LC followed by MS/MS for protein detection and quantitation. This multiplexed high-throughput proteomics approach enabled identification of 533 and 168 common proteins in the CE and MVs, respectively, in all four *Ng* strains in biological replicate experiments. Strain FA1090 was arbitrarily selected as the reference strain for calculating the protein abundance. After applying rigorous criteria, Zielke et al. (63) eliminated up to 68% of identified proteins. Among these proteins, 305 and 46 were uniformly present in the CE and MVs, respectively, in four strains. A total of 22 proteins were present at different levels in both analyzed subproteomes of these strains. Overall, these studies led to identification of a plethora of proteins that were either novel or had not been characterized in *Ng*. In this group were ubiquitous proteins localized to the CE and MVs: LPS-transport protein LptD (OstA, Imp), BamA, BamE, a predicted extracellular protein NGO1063 (SliC), and outer membrane proteins NGO1205 (ZnuD), NGO1344 (AsmA), NGO1956 (TamA), NGO1985 (BamG), NGO2111 (Slam2), NGO2121 (MlaA), NGO2139 (MetQ), and NGO2054 (63, 64). We focus on these proteome-derived antigens in the later sections of this article.

In the second proteomics-driven antigen mining approach for gonorrhea vaccine(s), our group was interested in discovering antigens induced in response to host-relevant environmental stimuli as they may represent novel protective antigens in distinct niches in the human host (64). Therefore, the model *Ng* strain FA1090 was subjected to aerobic (SG) and anaerobic (–O_2_) conditions, iron deprivation (–Iron), and exposure to normal human sera (+NHS; Figure 1), followed by CE protein extraction, trypsinization, iTRAQ labeling, and 2D-LC MS/MS (64). Three biological experiments yielded 751 common proteins with 17, 32, and 367 proteins with altered expression compared to SG in the presence of NHS, upon iron deprivation, and during anaerobic growth, respectively. In addition, 259 proteins were ubiquitously expressed under all conditions. There were many newly identified ubiquitous and differentially expressed CE proteins and potential new antigens including Slam2 and NGO1688 (both positively regulated by low iron), and ZnuD, which was induced under oxygen limitation (64).

In addition to the aforementioned approaches, we applied classical immunoproteomics to identify potential cross-reactive antigens in native MVs derived from *Ng* FA1090 that were intravaginally inoculated concurrently with interleukin-12 (Figure 1) and showed protection against heterologous *Ng* strains in the female mouse model of lower genital tract infection (52). Our approach relied on 2 DE SDS-PAGE separation of *Ng* native MVs coupled with immunoblotting with sera from MV-immunized mice (Figure 1). The overall MV proteome maps were created by staining proteins in a fluorescent stain. After the superimposition of antigenic maps (2D Immunoblot) with proteome maps, matching spots were excised and the proteins were subjected to trypsin digestion and MS/MS-based identification. The blotted protein maps consistently showed two spots of masses corresponding to 45 kDa and 43 kDa that were identified by MS/MS as translation elongation factor-Tu (EF-Tu) and a putative periplasmic polyamine-binding protein, PotF3, respectively. Supporting these findings, both proteins were also identified in our quantitative proteomic profiling of MVs derived from four common gonococcal isolates (63).

## Bioinformatics for Gonorrhea Vaccines

**Figure 2:**
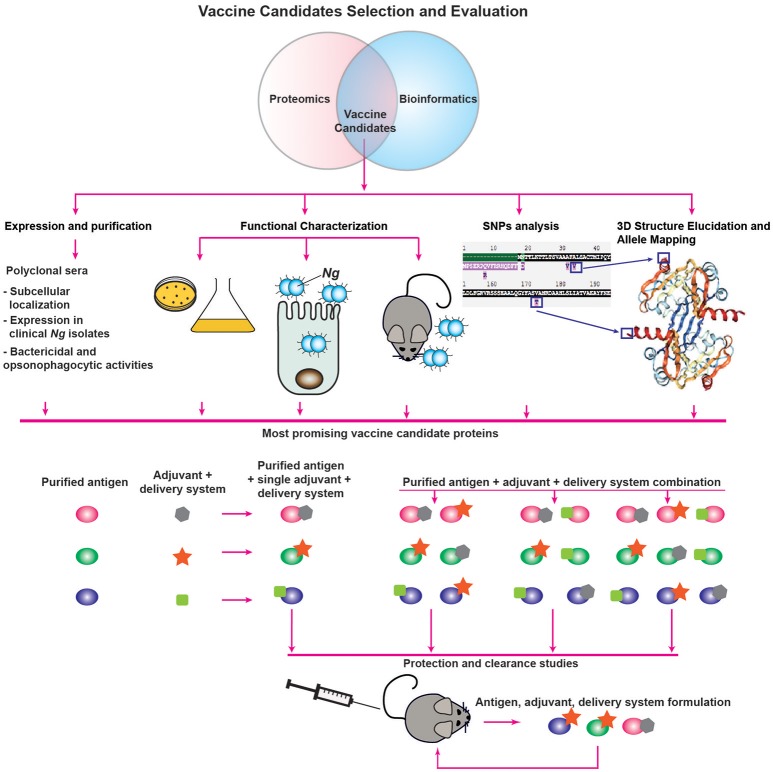
Vaccine candidate decision tree. Description is provided in the main text.

**Figure 3:**
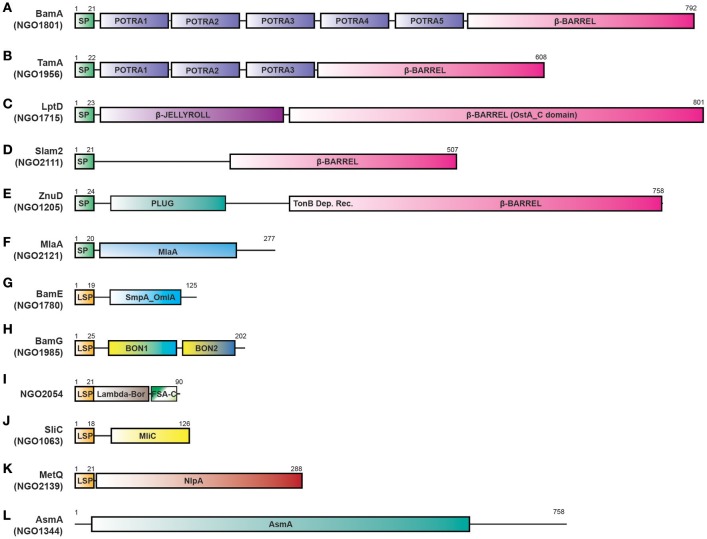
Proteome-derived antigen domain schematics. ORFs were examined for the presence of domains using KEGG, NCBI, UNIPROT, and literature searches. The presence of signal peptides was determined using the SignalP 4.1 and LipoP 1.0 servers. **(A)** BamA with a signal peptide, five POTRA domains, and an OMP β-barrel domain. **(B)** TamA protein with a signal peptide, three POTRA domains and an OMP β-barrel domain. **(C)** LptD with a signal peptide, N terminal β-JELLYROLL domain, and β-barrel OstA_C domain. **(D)** Slam 2 with a signal peptide and C terminal β-barrel domain (a DUF560 domain). **(E)** ZnuD comprises a signal peptide, plug domain, a Ton-B dependent receptor domain, and an OMP β-barrel domain. **(F)** MlaA with a signal peptide and predicted MlaA domain. **(G)** BamE containing a lipoprotein signal peptide and SmpA_OmlA domain. **(H)** BamG is composed of a lipoprotein signal peptide and two BON domains. **(I)** NGO2054 contains a Lambda-Bor domain that overlaps with a lipoprotein signal peptide, as well as a Fragile Site Associated protein C-terminus (FSA-C). **(J)** SliC containing a lipoprotein signal peptide and MliC protein family domain. **(K)** MetQ comprises a lipoprotein signal peptide and a NlpA domain **(L)** AsmA includes an AsmA domain. Schematics are not to scale. SP, signal peptide; LSP, lipoprotein signal peptide; BON, bacterial OsmY and nodulation; FSA-C, Fragile site-associated protein C-terminus; OMP, outer membrane protein.

After the mining and discovery of potential new antigens, insights can be gained into their suitability in a vaccine formulation through bioinformatic analyses to predict their function, subcellular localization, and post-translational modifications (Figure 2). In our studies, we employed PSORTb (87, 88), SOSUI-GramN (89), and CELLO (90, 91) algorithms to infer protein localization within the cell and to identify proteins discovered in the CE or MVs that localized to the outer membrane (63, 64). While each method considers the physiochemical properties of the amino acids that make up the protein, follow up computations differ between algorithms. To increase the accuracy of the subcellular localization predictions in our studies, we utilized a majority-votes strategy in which proteins were assigned to cellular compartments based on the results of at least two of the three methods (63, 64). To provide additional confidence in the localization predictions, searches for signal sequences recognized by signal peptidase (SPase) I or SPaseII can also be performed. The SignalP 4.1 server^1^ can be used as a tool to detect signal peptides (SP; Figure 3) associated with targeting to secretory pathways and cleaved by SPaseI (92, 93), while the LipoP 1.0 server^2^ detects lipoprotein signal peptides (LSP; Figure 3) associated with cleavage by SPaseII and further processing through the lipoprotein maturation pathway (94). Lipoproteins are characterized by an invariant cysteine residue that is modified with two or three acyl chains, which allows a hydrophilic protein to remain anchored to the membrane (95). Depending on secondary sorting signals, which are not well understood, lipoproteins may be anchored to either the inner or outer membrane and may face the periplasm or the extracellular milieu. Surface-exposed lipoproteins may be involved in nutrient acquisition, cell wall homeostasis, and adhesion to host cells (95), properties which we will discuss in more detail below. Finally, lipoproteins exposed to the extracellular milieu act as ligands for Toll-like receptor (TLR) 2 and may thus contribute to activating an adaptive immune response (95), a feature which makes them intriguing targets for vaccine development.

An additional analysis that can be performed to complement the prediction of subcellular localization includes functional category predictions through searches for Clusters of Orthologous Groups (COG). This analysis involves a phylogenetic comparison of the proteome-derived vaccine candidates to a database of proteins with known or predicted functions to determine to which cellular process(es) the protein is likely to contribute (96, 97). The results are grouped into four broad categories—Cellular Processes and Signaling, Information Storage and Processing, Metabolism, and Poorly Characterized—each of which can then be broken down further. Altogether, proteins can be clustered into one or more of 25 categories. Although the NCBI COG database^3^ does not contain information for *Ng*, COG data is available for *Nm*, which allows for functional analysis using a closely related organism. Insights gained into the function of the proteome-derived vaccine candidates will enable a more rational vaccine design approach, in which candidate antigens involved in multiple functions or in functional categories critical to bacterial fitness can be preferentially chosen in the vaccine decision tree and evaluated for their protective capabilities (Figure 2).

The proliferation of genomic data also benefits proteomics-driven reverse vaccinology. Of particular utility to gonorrhea vaccine research is the publically available *Neisseria* Multi Locus Sequence Typing database^4^ [*Neisseria* PubMLST, developed by Keith Jolley and sited at the University of Oxford; (98)], which has collected whole or partial genome sequence data from nearly 47,000 *Neisseria* isolates as of August 19, 2018. Annotation of the *Neisseria* pan-genome is underway, which would further facilitate vaccine development by identifying antigens that are present across the known population of *Ng* isolates, as well as proteins that are uniquely associated with highly antibiotic resistant strains. The wealth of information available from this database enables bioinformatic analyses of antigen conservation across numerous sequenced isolates. For example, in our characterization of a novel surface exposed lysozyme inhibitor of C-type lysozyme encoded by the *ngo1063* open reading frame, SliC, use of the *Neisseria* PubMLST database revealed the existence of only 10 closely related alleles with 9 single nucleotide polymorphic sites across nearly 5,000 *Ng* isolates. Further, 98% of isolates in the database possessed a single *sliC* allele (99), indicating that this protein is highly conserved and its inclusion in a vaccine could provide broad protection without the need for multiple antigenic variants.

Another analysis made possible by the information present in the PubMLST database is to map polymorphisms to available structural data (Figure 2). If highly prevalent polymorphisms are found on surface-exposed portions of the proteins, multiple recombinant proteins that incorporate the most common variants may need to be included in a vaccine to provide protection against a broader range of strains. While structural data will not necessarily be available immediately for all vaccine candidates, another computational analysis method may still enable prediction of surface exposed polymorphisms. Prediction of transmembrane helices by hidden Markov modeling using the TMHMM 2.0 server^5^ can reveal protein regions predicted to be internal, transmembrane, or external (100). Combining data from PubMLST with the results of TMHMM predictions can suggest which polymorphisms are likely to be surface exposed. Importantly, this analysis must be accompanied by surface-exposure assessments, as we discuss below, and may not be accurate for lipoproteins where the lipid anchor could be the only portion of the protein embedded in the membrane.

Additionally relevant to vaccine research are bioinformatic tools designed to predict the immunogenicity and protective capability of candidate antigens. Depending on whether the desired immune response is humoral (B-cell mediated) or cellular (T-cell mediated), numerous tools exist to predict whether antigen-derived peptides possess epitopes that are likely to be recognized by either of the major histocompatibility complex (MHC) class I or II proteins, the transporter associated protein (TAP) responsible for translocating peptides across the endoplasmic reticulum to MHC molecules for surface display, or B cell receptors. MHC I predictive tools have been estimated to be 90–95% accurate, although MHC II predictions are less reliable. Structural information is required for accurate B cell receptor binding predictions, as B cells and antibodies recognize the protein's native form. Certain tools use biochemical properties of the protein's constituent amino acids to predict likely B cell receptor binding sites. More accurate techniques require the protein's 3D structure as input [prediction tools extensively reviewed in (101)]. These prediction strategies may be useful to filter out antigens that are not likely to generate an immune response. However, follow-up immunological testing in the mouse as described below will be required to establish whether the response is protective or whether, as in the case of Rmp, the immunogenic response actively blocks the action of the adaptive immune system (33, 34).

## Vaccine Decision Tree

Subsequent to proteomic identification and bioinformatic analysis, candidate antigens should be assessed for their suitability for inclusion in a vaccine. Here, we suggest a decision tree for the evaluation of vaccine candidate proteins (Figure 2). To determine the expression characteristics of each vaccine candidate, they should initially be expressed and purified from a heterologous host, such as *E. coli*, and used to immunize rabbits or mice to collect polyclonal immune serum specific to the protein under investigation. These sera can then be used for several informative studies, such as confirmation of localization predictions by probing subcellular fractions to determine in which compartment(s) the protein is predominantly located. Immune sera can be used to interrogate the abundance of proteins after proteolytic shaving of intact cells, an experiment which will demonstrate whether proteins are accessible to external proteases (64). Protein accessibility to antibodies can also be directly investigated by immunoblotting analysis of intact cells spotted onto membranes and comparison of the signal to lysed cells using known surface-exposed proteins as controls (64). Antisera generated against vaccine candidates coupled with fluorescently labeled secondary antibodies can also be used to establish antigenic surface exposure through fluorescence-activated cell sorting analyses (68). Furthermore, primary antisera coupled with gold nanoparticle-labeled secondary antibodies can be employed in electron microscopy studies of surface exposure. With this type of study, not only can surface exposure be confirmed, but the localization, distribution and overall abundance of surface proteins can also be directly observed (102, 103). Serum raised against a candidate antigen additionally enables studies to accompany conservation predictions performed during initial bioinformatic analyses. In our assessments of candidate antigens, we employed a panel of at least 36 genetically, geographically, and temporally distinct *Ng* isolates, including the 2016 WHO reference strains (104), to examine whether the expression of each antigen is consistent across heterogeneous strains, as well as to determine whether the epitope(s) recognized by the antiserum are conserved in diverse gonococci and meningococci (64, 79, 99). Finally, to determine the likelihood that immunization with vaccine candidate antigens will be protective, antiserum raised against each antigen can be used to assess the protein's ability to elicit bactericidal and/or opsonophagocytic antibodies (24, 43). As mentioned previously, it is unknown whether antibodies' bactericidal or opsonophagocytic properties will predict their protective capabilities (24, 41). However, one or both characteristics is likely to contribute to a productive adaptive immune response, so this examination is useful in the absence of established protective mechanisms.

Studies that can be performed in tandem with antiserum-enabled investigations involve characterizations of the antigens' function(s) in gonococcal physiology and pathogenesis (Figure 2). We discuss these investigations in greater detail in a later section. In broad terms, the experiments associated with functional characterization are designed to assess the effects of conditional or isogenic knockout mutations on the ability of *Ng* to thrive *in vitro* under standard conditions as well as during exposure to stimuli relevant to infection, its ability to adhere to and invade epithelial cells, and its ability to colonize female mice during experimental infection in the murine model of lower genital tract infection or transgenic mice (41, 42). The overarching goal of these experiments is to determine the effects of the loss of each vaccine candidate, as antibodies are known to block protein function. In *Ng*, neutralizing antibodies against the nitrite reductase AniA inhibited its enzymatic activity (105). Neutralizing antibodies were also demonstrated to prevent MetQ-mediated gonococcal adhesion to epithelial cells (106), and were able to abrogate *in vitro* lysozyme inhibition by the *Neisserial* adhesin complex protein (ACP), a dual-function protein involved in adhesion and defense against lysozyme attack (99, 107–109). Additionally, *Nm* and *Ng* IgA1 protease-mediated IgA cleavage was inhibited by sera collected from both acute and convalescent meningitis patients (110). More specifically, antibodies that block protein function are elicited by immunization with each of the recombinant protein components of the 4CMenB vaccine—factor H binding protein (fHbp), Neisserial heparin binding antigen (NHBA), and *Neisseria* adhesin A (NadA). Antibodies against fHbp abrogate factor H binding and thus enhance meningococcal serum sensitivity (111). Both NHBA and NadA are involved in bacterial adherence, and neutralizing antibodies against either protein reduce the ability of *Nm* to adhere to epithelial cells (112, 113). Non-neutralizing antibodies provide protection against viruses (114, 115); however, similar data are scarce for bacteria (114). Targeting bacterial virulence factors or physiologically important proteins with a vaccine could discourage mutations that would allow the protein to evade immune detection, thereby improving the vaccine's success. The exclusive focus on targeting virulence factors in a vaccine is not vital, however, as demonstrated by immunization with the *S. agalactiae* surface immunogenic protein (Sip), which induces a strong protective response (116) but has no reported function in the pathogenesis of group B streptococci. It is possible that the antigens eventually formulated into a successful gonorrhea vaccine will not generate neutralizing antibodies, but evidence suggests antibodies that block protein function to some extent are elicited in the majority of immune responses effective at protecting against bacterial pathogens.

An additional study that can be used to inform vaccine design involves antigen structural elucidation, either alone or in complex with antibodies directed against the target protein. We discuss structural studies that have been performed on proteome-derived vaccine candidates in a subsequent section. Not only does the structure of a protein give insights into its function, but it also enables allele mapping, as discussed in the previous bioinformatics section (Figure 2). Traditionally, structural characterization is performed through X-ray crystallography (117), although advances in nuclear magnetic resonance spectroscopy also allow for the structural elucidation of small proteins (118, 119). Co-crystallization with antibodies can reveal the mechanism(s) of action of the antibodies against the target protein (120–122). This strategy requires the use of antigen binding fragments of monoclonal antibodies (mAbs), which are more technically challenging, time-consuming, and expensive to produce than polyclonal antibodies (123). For this reason, co-crystallization is likely to be pursued only after further evaluation of the antigen's immunogenicity and protective capabilities as part of a vaccine. A complementary technique that can be pursued for difficult-to-crystallize antigen/antibody complexes is cryo-electron microscopy, in which single molecules embedded in a flash-frozen matrix are visualized with an electron microscope. This technique was successfully employed to elucidate the structure of an integral membrane ion channel protein (124), as well as the interaction interface between an antibody fragment that successfully neutralized a range of influenza virus variants and the receptor site of the influenza virus hemagglutinin protein (125). One limitation of cryo-electron microscopy is that structural resolution tends to be poorer than with the use of other techniques. However, recent technical advances have been able to improve acquired structures to near-atomic resolution [<4Å; (126)]. Although cryo-electron microscopy may circumvent some limitations of X-ray crystallography or nuclear magnetic resonance, mAbs will still be required for evaluating antigen/antibody interactions.

Finally, if the results of the evaluation studies suggest that a protein may be a suitable vaccine component, its immunogenicity and ability to elicit a protective response will be investigated through protection and clearance studies performed in the female mouse model of gonorrhea (Figure 2). To date, only an extremely limited number of studies have been published examining the ability of vaccine formulations to accelerate clearance and protect against subsequent infection in the mouse model. These studies include intranasal immunization with MVs, which was associated with enhanced clearance in one study (127), but not in another (24). However, intravaginal immunization with MVs combined with microencapsulated IL-12 both accelerated gonococcal clearance and protected mice from subsequent infection (52). Active intraperitoneal immunization with a peptide mimic of a conserved LOS epitope recognized by the 2C7 mAb or passive immunization with the mAb itself both shortened disease duration (128). Finally, mice immunized in the rear footpads with viral replicon particles (VRPs; viral derivatives that deliver antigens after a single replication cycle) loaded with the outer membrane porin PorB, combined with a subsequent booster immunization comprised of recombinant refolded PorB and an adjuvant, cleared experimental infections significantly more quickly than control mice (24). Follow up experiments suggested that this protection was likely associated with the adjuvant effect of the VRP itself, rather than a specific protective effect of the PorB antigen (24). These studies, and their paucity, reveal the need to accelerate and expand systematic immunization studies that examine the protective effects of antigens individually and in combination. Additionally, the effects of the presence or absence of adjuvants with each antigen formulation should be examined, as different adjuvants can drive specific adaptive immune responses (Figure 2). Currently, vaccines that induce a balanced Th1/Th2 responses are considered optimal [reviewed in (129)] and are important in defending against gonorrhea (52, 53). Examples of FDA-approved adjuvants that should be evaluated based on these considerations include alum, which drives a Th2 response; monophosphoryl lipid A, a detoxified LPS derivative that acts as a TLR4 agonist to stimulate a Th1 response; and oligonucleotides enriched in cytosine phosphoguanine (CpG) islands (129, 130), which are TLR9 agonists that mediate Th1 and CD8 cellular immune responses but also increase antibody titers (131). To optimize the chance of developing an effective vaccine, the route of immunization, as well as different antigen delivery systems that potentiate immune responses, should be considered and investigated. Numerous delivery systems are in clinical and pre-clinical stages of development, including virus-like particles, emulsions, liposomes, polymer-based systems, hydrogels, and implants [reviewed in (129, 130)]. Finally, evaluation of subunit vaccines beyond their ability to accelerate clearance and protect against subsequent infection will include quantification of antigen-specific IgG and IgA antibody titers in serum and vaginal mucosal secretions, evaluation of antibodies' bactericidal and opsonophagocytic activities, and examination of the cellular immune response.

While we recognize that the decision tree presented here may appear daunting, it is important to remember that traditional vaccinology has failed to deliver a successful gonorrhea vaccine. Development of new vaccines is not trivial. The challenges inherent in protecting against this highly adaptable pathogen necessitate creative and flexible approaches. The decision tree and the generation of effective gonorrhea vaccines will be shaped by lessons learned from the *Ng* biology and epidemiology as well as new delivery systems and technologies. A strength of our proposed decision tree is that, regardless of whether an antigen is ultimately formulated into a vaccine, more information will be gained into *Ng* pathophysiology and the gonorrhea research field will be accelerated.

## Elucidation of Function of the Proteome-Derived Vaccine Candidates

We propose a vaccine-induced immune response that targets antigens important for CE homeostasis, bacterial pathogenesis, or overall viability may not only protect against the acquisition of subsequent gonorrhea infections but may also enhance immune system efficacy by weakening the gonococcus, thus accelerating the clearance of ongoing infections. A vaccine that employs this strategy could conceivably be used for therapeutic interventions in addition to preventative purposes. Studies evaluating the effectiveness of a vaccine against herpes simplex virus type 2 (HSV-2) provide a precedent for a therapeutic vaccine. In both guinea pigs (132) and humans (133, 134), vaccination against HSV-2 tended to decrease the lesion rate and reduced viral shedding for at least 12 months—an evaluation criterion for efficacy of HSV-2 antiviral agents (134). A neutralizing antibody response was key for the vaccine's immunotherapeutic activity (132). For this reason, determining the function of antigens proposed for inclusion in a vaccine is useful to predict the potential physiological effects of an immune response that blocks protein function. The scientific literature contains a wealth of information that can be used as a starting point to facilitate functional characterization. Numerous gonococcal proteins have homologs that have been investigated in *E. coli* or *Nm*. However, these studies should be approached with a modicum of caution, as protein function may differ between species. For example, we determined that LptD is essential in *Ng* (63), similar to findings in *E. coli* (135, 136). In contrast, this protein is dispensable for *Nm* (137). Furthermore, fHbp, which is a surface-exposed protein that contributes to *Nm* serum resistance, has no signal peptide for outer membrane localization in gonococci, nor does loss of *Ng* fHbp alter bacterial susceptibility to human serum (138). The potential for distinct protein function, even among closely related species, emphasizes the importance of performing independent studies to examine the role of homologous proteins in the organism being targeted by the vaccine.

A common tool for the study of protein function is a bacterial strain with a knockout mutation in the protein of interest. This can be accomplished through homologous recombination-mediated allelic replacement of the genetic locus with an antibiotic resistance marker, as we have performed in our studies (63, 64, 99), or through gene inactivation by targeted transposon insertion mutagenesis (139). Of course, attempting to knock out an essential gene with this strategy will be unsuccessful, as transformation efforts will not result in any colonies, or off-target mutations may occur that lead to antibiotic resistance but do not affect the target gene. Neither outcome is desirable. Our strategy to circumvent this difficulty has been to place the gene of interest at an unlinked locus under the control of an inducible promoter, then to replace the native gene with an antibiotic resistance cassette while inducing protein expression from the heterologous locus (64, 140, 141). With this technique, the effects of protein depletion, as well as protein stability, can be studied for essential genes. Using knockout mutant strains of non-essential genes, protein function can be assessed by exposing bacteria to different stress conditions and monitoring for growth. We evaluate bacterial survival under conditions relevant to human infection, including iron starvation, exposure to human serum, and anoxia, with the hypothesis that bacteria deficient in proteins important to a rapid or appropriate response to any of the conditions will be non-viable or will not grow as robustly as wild type bacteria (79, 99, 142). CE permeability and stress can be evaluated by exposure to different antibiotics, either with the use of Etest antimicrobial test strips^6^ or serial dilutions of bacteria inoculated onto solid medium supplemented with antibiotics (63, 79, 99). Additionally, overproduction of MVs is a general marker of CE stress (143–145), so a comparison of MV production between wild type and mutant bacteria can suggest the level of CE stress resulting from the loss of a protein (79). Qualitative or quantitative proteomic profiling of supernatants collected from liquid cultures can give insights into the extent of membrane leakage or cellular lysis associated with deficiency of each vaccine candidate (146, 147). Finally, *in vitro* tissue culture experiments can inform whether the protein under investigation is involved in, or has substrates that contribute to, the ability of *Ng* to adhere to, invade, and survive within human cervical epithelial cells (99, 106, 148, 149).

An innovative technique that we adapted for the first study of its kind in *Ng* is the use of phenotype microarrays [PMs; (147)]. Developed by Biolog^7^, each PM is a 96-well microtiter plate pre-formulated with varying concentrations of numerous diverse compounds to assess bacterial nutritional requirements and sensitivity to chemical agents, including antibiotics and osmolytes (150, 151). *Ng* nutritional requirements are well established. Glucose, pyruvate, and lactate are the only carbon sources the gonococcus is able to utilize (152). Therefore, we focused solely on chemical sensitivity PMs to assess the ability of seven vaccine candidates to defend against osmotic shock, as well as their physiological roles during exposure to CE-perturbing agents including metals, antimicrobial peptides, small hydrophobic molecules, and dyes (147). In our investigation with a comprehensive screen of over 1,000 conditions, we discovered 323 conditions that affected at least one of the mutant strains tested. Using these data, we generated a dendrogram based on the similarity between the effects of the loss of each protein, which revealed that the defects associated with knockout mutations of BamG or MlaA were the most distinct from the other five strains tested. The results of PM screening suggested these two antigens would be the most suitable to include in a vaccine, due to the extensive chemical sensitivities associated with the loss of either protein (147).

Although *in vitro* experiments are useful for predicting protein function, it is impossible to simultaneously account for all factors that will be encountered during infection of the host. For this reason, infection studies in the female mouse model are invaluable (Figure 2). Experimental infections with a single strain can be used to determine whether the bacterial load or infection duration is altered in the absence of a target protein (42, 153). Alternatively, competitive infections between the mutant strain and wild type bacteria can be performed to minimize mouse-to-mouse variation and to directly associate fitness phenotypes with the loss of a protein (153). Finally, to determine whether the protein under investigation contributes to bacterial response to hormones, ovariectomized mice can be utilized to decrease hormonal influence over infection characteristics (43, 153).

The studies outlined above will facilitate vaccine development by identifying antigens with roles in maintaining gonococcal fitness under stress conditions, including during active infections.

## Function of Proteomic-Derived Antigens in CE Homeostasis and Nutrient Acquisition

**Figure 4:**
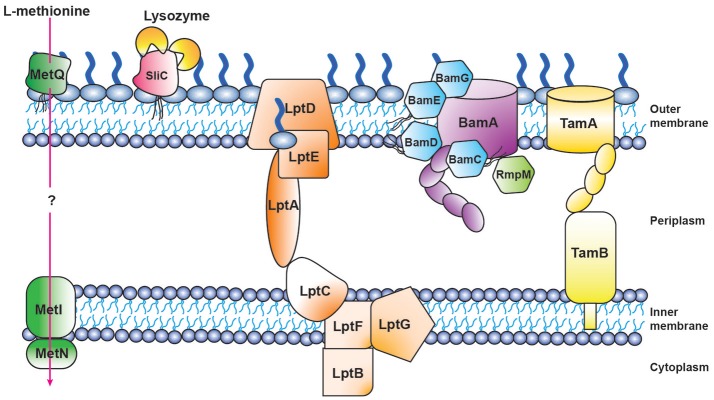
Schematic of proteomics-derived antigens MetQ, SliC, LptD, BAM, and TAM function-structure and organization in *Neisseria gonorrhoeae*.

In the following sections, we discuss insights into function-structure of 12 proteomics-derived antigens (Figure 3). We have already verified surface-exposure for a majority of these vaccine candidates, as well as their expression and conservation among diverse *Ng* isolates. We have also established that BamA, TamA, LptD, MetQ, and NGO2054 elicit bactericidal antibodies that cross-react with heterologous *Ng* strains (64). Our initial characterization of these vaccine candidates in *Ng*, in addition to studies of the functions of homologous proteins in *Nm* show that many of them play different roles in the CE homeostasis or in nutrient acquisition. Maintaining the integrity of the OM, as well as acquiring nutrients, are critical for bacterial survival in hostile environments, such as those encountered during infection of the host. As a gram-negative bacterium, *Ng* possesses a typical CE, composed of a cytoplasmic or inner membrane, a cell wall made up of peptidoglycan, and an asymmetric OM (Figure 4). The outer membrane of *Neisseria spp* contains LOS, rather than the more typical LPS, which reflects their niche as mucosal pathogens (154). LOS acts as a buffer region for protection against environmental insult and contributes to pathogenesis through several mechanisms (154, 155).

Three of our antigens (BamA, BamE, and BamG) are components of the β-barrel assembly machinery (BAM) system (63, 79, 156), which is responsible for folding and inserting β-barrel proteins into the bacterial outer membrane (157, 158). Until recently this critical protein complex has been primarily investigated in *E. coli*, where it consists of the central β-barrel “Omp85” protein BamA, assisted by four lipoproteins (BamB-E). In contrast to *E. coli*, gonococcal BAM lacks BamB and contains a surface-displayed BamE [Figure 4; (79)]. Further, *Neisseria* possess an additional non-essential accessory protein RmpM (159). Similarly to other gram-negative bacteria, BamA and BamD are essential for *Ng* cell viability, and depletion of either BAM component results in OMP misfolding, as well as defects in protein stability and assembly (64, 160–162). BamA is responsible for folding and inserting virtually all β-barrel OMPs in the OM (158). In contrast, BamE is not essential, which enables study of its effects on the CE. Our comprehensive analyses, including PMs, showed that the loss of BamE renders *Ng* susceptible to a wide range of compounds including detergents, antimicrobial peptides, other membrane-perturbing agents, and antibiotics (79, 147). Enhanced production of MVs is a marker of CE stress (143). Accordingly, the Δ*bamE* mutant produced significantly increased amounts of MVs in comparison to WT bacteria. Additionally, the MV protein profile exhibited distinct alterations, suggesting that certain proteins were specifically targeted for packaging into MVs (79). Finally, removal of BamE appeared to destabilize the BamA-D interaction, which resulted in BamD localized on the cell surface of BamE-deprived *Ng* (79). The aberrant BamD localization in the absence of BamE could further increase membrane perturbations by interference with proper OMP insertion by BamA.

We recently discovered that one of our proteome-derived vaccine candidates, NGO1985, is a previously unrecognized accessory lipoprotein within the BAM complex and therefore renamed this protein BamG (156). Included in BamG is a lipoprotein signal peptide, which targets the protein for surface display (156), as well as two bacterial OsmY and nodulation (BON) domains (Figure 3H). BON domains are proposed to bind phospholipids based on the finding that *E. coli* OsmY prevents the inner membrane from shrinking during osmotic shock, although this hypothesis was not experimentally verified (163–165). In *Ng*, our initial probing with detergents and PM analyses revealed an extensive sensitivity phenome associated with lack of BamG, suggesting that BamG, through its interaction with the BAM complex, plays a critical function in CE biogenesis (63, 147, 156).

LptD belongs to the low abundant, large and complex class of BAM substrates that are also heavily dependent on SurA, Skp and FkpA chaperones (166). LptD, together with OM-localized LptE (Figure 4), are constituents of the lipopolysaccharide transport (LptA-F) complex, and are crucial for inserting LPS into the outer leaflet of the *E. coli* outer membrane (167). The Lpt system appears not to function in a completely conserved manner in gram-negative bacteria, as both *Ng* and *E. coli* LptD are essential (63), whereas *Nm* can survive without LOS and none of the Lpt components are important for bacterial viability (168).

For the bacterial CE to exert its barrier function, the outer leaflet of the outer membrane must be a homogeneous layer of LOS. If this LOS layer is disrupted, phospholipids may diffuse from the inner leaflet and weaken the barrier function of the outer membrane. The Mla (maintenance of lipid asymmetry) system, which is conserved across gram-negative bacteria and is composed of MlaA-F, removes misplaced phospholipids from the outer leaflet and re-integrates them into the inner membrane to mitigate the detrimental effects of altered membrane asymmetry. The outer membrane component of the Mla system, MlaA, has primarily been studied in *E. coli*, where its deletion enhances bacterial susceptibility to membrane perturbing agents (169). In *Ng*, MlaA is encoded by the *ngo2121* locus and comprises a signal peptide and a MlaA domain (Figure 3F). Gonococcal MlaA is annotated as a lipoprotein, although we have previously noted that it does not contain the invariant cysteine residue required for lipid modification (147). However, a lipoprotein signal peptide is present in homologs from *E. coli, K. pneumoniae*, and *S. marcescens* (169, 170). We are currently investigating the implications of the lack of MlaA lipidation in *Ng*. Combining antibiotic susceptibility testing using Etests with PM screening, we have established that compounds targeting the CE, including antimicrobial peptides, are more effective against *Ng* lacking MlaA than wild type bacteria. Resistance to antibiotics acting against cytoplasmic targets was unaltered (63, 147, 171), suggesting a specific outer membrane defect (169). Additionally, the Δ*mlaA* knockout showed increased vulnerability to oxidative stress-inducing compounds (147) and produced more MVs than wild type bacteria (171). Together, these findings suggest that MlaA is required for maintenance of outer membrane homeostasis and is involved in MV biogenesis (172).

Bacterial lipoproteins play numerous roles in cellular physiology, adhesion to host cells, modulation of inflammatory processes, and transport of virulence factors into host cells. Proper lipoprotein localization is critical for protein function (173). The proteome-derived vaccine candidates BamE, BamG, SliC, MetQ, and NGO2054 are newly described *Ng* lipoproteins (Figure 3). NGO2111 is homologous to the *Nm* surface lipoprotein assembly modulator Slam2, which is involved in translocating the hemoglobin-haptoglobin utilization protein to the cell surface (174). Slam2 is highly conserved amongst *Neisseria* isolates and is not found in *E. coli* (175, 176). *Ng* Slam2 contains a signal peptide and a 14-stranded β-barrel domain, which has also been annotated as a DUF560 domain (Figure 3D; 147, 176). In our PM study, we found no conditions that uniquely affected a Δ*slam2* mutant, although 36 compounds were either beneficial or detrimental to the growth of this strain, in common with one or more of 6 other mutants (147). These results suggested that Slam2 exerts an indirect effect on CE integrity, potentially through an undiscovered lipoprotein substrate.

Another gonorrhea vaccine candidate we investigated in our PM study was NGO1344, which contains an AsmA domain (Figure 3L) and is homologous to the AsmA protein from *E. coli* and *Nm*. In *E. coli*, loss of AsmA decreased LPS synthesis (177); however, a similar phenotype was not observed in a *Nm* Δ*asmA* mutant (178). Our analysis of cell lysis indicated that loss of *Ng* AsmA resulted in elevated levels of several cytoplasmic proteins in culture supernatants, especially when grown in the chemically-defined Graver-Wade liquid medium (147). Fifty-three membrane-perturbing compounds affected the viability of a Δ*asmA* mutant, including polymyxin B and bile salts (63, 147). Clustering analyses suggested that the loss of AsmA resulted in phenotypes similar to those observed upon BamE deletion (147). Our results indicate that gonococcal AsmA contributes to CE integrity.

We also examined the role of the small lipoprotein NGO2054 in CE homeostasis. This protein is comprised of a lipoprotein signal peptide, a Lambda-Bor motif that contributes to serum resistance in *E. coli* (179), and a region with homology to the C-terminus of the fragile site-associated protein involved in adipocyte differentiation in mammalian cells [Figure 3I; (180)]. A Δ*ngo2054* mutant was the least affected in our PM screen; only 27 compounds altered its growth (147). Thus, although we have established that NGO2054 is surface exposed, well conserved, and elicits bactericidal antibodies (64), its function within the CE remains enigmatic.

Metal co-factors such as zinc and iron are critical to facilitate cellular and enzymatic processes within pathogenic bacteria. During infection, bacteria rely on scavenging these metals from the host. To combat against bacterial pathogens, host organisms sequester available metals at the site of infection, a strategy termed nutritional immunity. The *Neisserial* outer membrane zinc uptake protein ZnuD overcomes host-imposed zinc depletion (181). In *Ng*, ZnuD is encoded by the *ngo1205* locus, which includes an amino terminal signal peptide, a plug domain, as well as a TonB β-barrel (Figure 3E). In support of its role as a zinc uptake protein, a *Nm* Δ*znuD* mutant was more sensitive to the bactericidal effects of neutrophil extracellular traps (182), which contain calprotectin that sequesters zinc ions (183). Additionally, meningococci deficient in ZnuD were attenuated in a systemic infection model (181), although Δ*znuD* gonococci exhibited no survival defect during intracellular infection of cervical epithelial cells (184). We performed our PM analysis on *Ng* Δ*znuD* bacteria to examine the contribution of ZnuD to CE integrity. Thirty-seven conditions altered the growth of bacteria lacking ZnuD, including the divalent cation chelator ethylenediaminetetraacetic acid (147). Our results provide support for the role of ZnuD as a zinc uptake protein and suggest that downstream effects to the CE occur from the decreased ability to acquire zinc.

Translocation of solutes across the CE is mediated by ABC transporters, which utilize the hydrolysis of ATP to transport molecules. A substrate binding protein is necessary to capture the substrate. Our proteome-derived vaccine candidate MetQ (63, 64), which was highly conserved in *Ng* as well as *Nm* (55, 64), is homologous to the *E. coli* methionine binding protein MetQ [also known as NlpA; (106)] and is annotated with a lipoprotein signal peptide and a NlpA domain (Figure 3K). The presence of an operon upstream from *metQ*, composed of an ATP-binding protein (MetN) and a transmembrane permease (MetI) (106), provides additional support for the role of MetQ as the substrate binding protein in an ABC transport system. Surface plasmon resonance experiments demonstrated that *Ng* MetQ binds L-methionine with nanomolar affinity (106). These findings, in combination with our experiments demonstrating that *Ng* MetQ is surface exposed, highly conserved, and elicits strongly bactericidal antibodies (64), suggest MetQ is a promising vaccine candidate. Targeting MetQ could interfere with methionine transport and result in downstream protein synthesis defects.

The studies described here illustrate the myriad cellular processes in which our proteome-derived vaccine candidates participate. A subunit vaccine composed of several of these antigens has the potential to compromise gonococcal fitness independent of factors beyond a neutralizing antibody response.

## Function of SliC and MetQ in Pathogenesis

Bacterial pathogenicity is influenced by the number of infecting bacteria, the route of entry, the presence of host defense barriers, and bacterial virulence factors. *Ng* pathogenesis involves a collection of factors: pili, Opa proteins, LOS, and peptidoglycan all contribute to infection and are important for optimal pathogenesis (6). The study of protein function has the potential to reveal previously unrecognized virulence factors. A perfect example is our discovery of the surface-exposed inhibitor of C-type lysozyme, SliC (99). Through bioinformatic analyses, we determined SliC, encoded by the *ngo1063* locus, contained a lipoprotein signal peptide and a domain similar to membrane-bound lysozyme inhibitors of C-type lysozyme (MliC domain; Figure 3J). Bacterial proteinaceous lysozyme inhibitors protect the cell wall against host lysozyme attack during infection (185). Lysozyme inhibitor proteins were not known to exist in *Neisseria* until the recent discovery that the *Neisserial* adhesin complex protein (ACP) is inhibitory toward human (HL) and hen egg white lysozyme (HEWL), both of which are C-type lysozymes (108). The activity of SliC as a lysozyme inhibitor was comprehensively examined using an assay that involved fluorescently labeled peptidoglycan. Pre-incubation of wild type SliC with HL or HEWL completely obstructed peptidoglycan hydrolysis. In contrast, SliC mutated in two residues predicted to be key for the protein's interaction with lysozyme (S83A and K103A) showed no inhibition of cell wall hydrolysis with the addition of HL or HEWL (99). Subsequently, bio-layer interferometry was used to assess the kinetic interaction between SliC and lysozyme. This analysis revealed moderate binding between SliC and HL *in vitro* (K_D_ 11 μM), with one SliC molecule binding two HL molecules [Figure 4; (99)]. Surprisingly, cells that lacked SliC showed no difference to wild type when exposed to increasing levels of HL *in vitro*. However, a double SliC/ACP knockout was strongly attenuated during HL exposure (99, 109), suggesting that one lysozyme inhibitor compensates for the lack of the other protein. Despite this compensatory activity, *Ng* bacteria lacking SliC were up to 250-fold less fit during a competitive infection with WT bacteria in the mouse model (99). Further, bacteria complemented with a SliC S83A/K103A mutant were also significantly attenuated during competitive infections (99). *In vitro* adhesion and invasion assays revealed no difference between Δ*sliC* and WT bacteria, which indicated that the attenuation observed in the mouse was not due to defects in the mutant's ability to adhere to or invade epithelial cells. Finally, Δ*sliC* bacteria were as fit as WT during competitive infections in a lysozyme-deficient mouse, which provided conclusive evidence that the inhibitory activity of SliC against host lysozyme is critical for gonococcal colonization (99).

Substrate binding components of ABC transporters may be localized to the outer surface and may contribute to cell adherence (186, 187). MetQ from *Ng* was therefore assessed for its ability to mediate bacterial adhesion and invasion of cervical epithelial cells. Gonococci lacking MetQ exhibited a 2.4-fold decrease in adherence and a 1.5-fold lower level of invasion compared to the wild type strain. These findings were comparable to the level of adherence and invasion within transformed primary cervical epithelial cells (106). Additionally, bacterial survival in primary monocytes and macrophages was evaluated, to determine whether MetQ exerts a protective role against immune cells or other similar factors. Cells lacking MetQ displayed a 2.3-fold reduction of viability in primary monocytes and a 1.5-fold decline in macrophages. MetQ-deficient gonococci were also significantly attenuated upon exposure to human serum (106). The results of this study indicated MetQ, in addition to its role as a methionine transport protein, contributes to the ability of *Ng* to adhere to and invade epithelial cells and protects against the innate immune system.

The study of vaccine candidates' contributions to bacterial pathogenesis benefits vaccine development by revealing potential vulnerabilities that can be exploited through rational vaccine design to cripple the invading bacterial pathogen.

## Structure of Proteomic-derived Antigens

Structure-based antigen design offers new possibilities in vaccine development and improvement by delivering novel immunogens and informing about protective epitopes (188). This approach, in combination with sequencing data and computational biology studies (189), can drive rational optimization of vaccines as we discussed above (Figure 2). In this section, we will focus on summarizing structural investigations that have been performed on our proteome-derived vaccine candidates. To this end, only the structures of *Ng* BamA and BamE have been solved (79, 190). However, *Nm* crystal structures of MetQ and ZnuD are available in addition to LptD, BamA, BamE, and TamA obtained from different bacterial species, all of which provide information about antigen architecture and conformational conservation.

The first BamA crystal structure was solved from *Ng* and revealed a 16-strand β-barrel domain within the outer membrane, connected to five N-terminal periplasmic polypeptide transport-associated (POTRA) domains [Figure 3A; (190)]. The BamA crystal structure from *E. coli* displayed a high level of flexibility between POTRA_5_ and the β-barrel domain, suggesting that the POTRA domains assist in transferring the substrate to the β-barrel domain of BamA (191). A prominent difference was found in BamA conformation in *Ng* compared to BamA in *Haemophilus ducreyi*, where the last β-strands within the barrel are tightly meshed with hydrogen bonds, providing more rigidity. However, in *Ng*, the last β-strand is bound to the first by only two hydrogen bonds, which allows the pore itself to twist. The POTRA_5_ domain of BamA in *Ng* is positioned closely to the barrel, with periplasmic loops 3, 4, 5, and 7 stabilizing the closed conformation. This is highly different in *H. ducreyi*, as the POTRA region hinges outwards ~70°, which does not allow POTRA_5_ to interact with the β-barrel periplasmic loops (190). The difference in the interaction between POTRA_5_ and the β-barrel may act as a secondary mechanism to prevent unregulated solute entry into the pore and may compensate for the lack of hydrogen bonding in *Ng* BamA. Further analysis of the *Ng* BamA crystal structure revealed the hydrophobic belt along the C-terminal strand was narrower (~9Å) than the opposite side of the barrel [~20 Å; (190)]. The authors of this study hypothesize that this reduced width may disrupt the lipid membrane environment and act to allow easier insertion of the OMP into the membrane (190). Based on the configuration of BamA, two hypotheses have been proposed for the mechanism of OMP folding and insertion, depending on the complexity of the substrate protein. Complex proteins take advantage of a lateral opening event facilitated by a conformational switch of loop 6 and the gating motion of the POTRA domains. The nascent OMP is threaded through the β-barrel and uses exposed strands of BamA as a template for proper barrel formation through a transient OMP-BamA complex until the new OMP buds off into the OM. Simple substrates may bypass the BamA β-barrel completely and may be inserted directly into the destabilized portions of the membrane through their interactions with the POTRA domains (190). Recent studies suggest that OMP binding to BamD induces conformational changes in the extracellular loops of BamA for substrate folding and membrane insertion (192). Thus, antibodies against these extracellular loops may interfere with proper OM biogenesis and cause severe downstream effects to OM integrity.

Gonococcal BamE includes a lipoprotein signal peptide, as well as a predicted SmpA_OmlA domain [Figure 3G; (193)] In *E. coli*, X-ray crystallography showed BamE interacts exclusively with BamA, and does not contact other accessory lipoproteins. Instead, BamE directly assists OMP folding through its interaction with BamA. In contrast to *E. coli*, where the native form of BamE appears to be a periplasmic monomer (194), *Ng* BamE is a surface-exposed dimer that also includes an additional C-terminal helix not present in other solved structures of BamE (79). Isolation of proteins from native membranes should definitively establish which conformation is the active state. The dimeric form of BamE possesses structural homology to β-lactamase inhibitors, which has led to the hypothesis that BamE has a secondary function as a β-lactamase inhibitor (195, 196). Surface-exposed dimers of BamE in *Ng* may therefore act as a first line of defense against β-lactam antimicrobials. In support of this secondary function of BamE, *Ng* Δ*bamE* mutants were more sensitive to several different β-lactam antimicrobials (79, 147). Further investigation will be required to determine whether BamE contributes to antibiotic resistance. If BamE does in fact possess dual functions, a neutralizing immune response could both interfere with OM integrity as well as enhance antibacterial efficacy.

Autotransporter biogenesis relies on a passenger-transport complex to assist in the translocation of autotransporters across the outer membrane. The translocation and assembly module (TAM) comprises a two-membrane complex containing a β-barrel OMP TamA and inner membrane protein TamB, a nanomachine required for virulence of pathogenic bacteria [Figure 4; (197)]. This complex facilitates the folding and insertion of the autotransporter domain and has been hypothesized to assist in proper effector domain folding. Effector domains are responsible for protein activity and frequently contribute to virulence (198). Consistent with its role in translocation, TamA contains a β-barrel structure homologous to BamA (199) and has been hypothesized to be the result of a duplication event that arose from the evolutionary convergence of BamA and TamB (200). In *E.coli*, the TamA structure was determined at 2.25 Å resolution, revealing a sixteen stranded β-barrel ring structure from amino acids 265-577 on the C-terminus, and three POTRA domains between amino acids 22-264 on the N-terminus [Figure 3; (201)]. The POTRA domains wind ~50Å, oriented toward the periplasm in a semi-circle arrangement (201). The initial contact between substrate and TamA is facilitated by interactions between TamB and the POTRA domains, which act as a hinge mechanism (197, 200). Additional crystal structure analysis showed a kink in the C-terminal β-strand pointing inward, which weakened the lateral wall, implying a possible gate for substrates to route toward the lipid bilayer (201). The kink resulted in a weak β-strand pair of strand 1 and 16, because of three main chain hydrogen bonds. The kink on β-strand 16 results in a large gap between the ends of strands 1 and 15 near the POTRA_3_ attachment site. This cleft promotes insertion of the substrate, mediated by interactions between the β-barrel and the POTRA domains. The folding and insertion process is hypothesized to begin when an autotransporter's β-barrel domain engages with POTRA_3_ within the periplasm and is guided toward the barrel of TamA. The structure of TamA also includes a closed extracellular lid from amino acids 456-495. Salt bridge interactions between the lid and the β-barrel are mediated by Arg477 in the lid and Asp521 in the barrel (201). Targeting the epitopes responsible for stabilizing the interaction between lid and β-barrel with an immune response could interfere with proper TamA function.

The crystal structures of the core LPS assembly LptDE protein complexes from several bacteria, including medically important pathogens, have been obtained, with only two full-length structures of *Shigella flexneri* and *Klebsiella pneumoniae* LptDE (167, 202). Overall, these studies revealed a strong structural conservation of the two-protein plug and barrel assembly and demonstrated that LptE was integrated within the 26-stranded, C-shaped β-barrel architecture of LptD. All LptDE structures have a negatively charged lumen which may facilitate LPS/LOS insertion (202). Additionally, LptD contains a periplasmic β-jellyroll domain, which is structurally similar to LptA and facilitates LPS/LOS transit through the periplasm [Figure 3C; (167)]. The N terminal domain of LptD undergoes a 21° rotation, which may aid assembly or influence flexibility of the LptCAD scaffold (202). The LptD β-barrel is large compared to other β-barrel OMPs. Its dimensions accommodate large substrates and facilitate efficient transport. Further, a kink was present in the first two β-strands of the β-barrel due to two proline residues in β1 and β2 (167), and they have been experimentally verified to play a pivotal function in the lateral opening of the barrel (202). The interaction between LptD and LptE is dependent on polar connections within the LptD β-barrel inner cavity; thus, LptE supports LptD structural maintenance, as well as export of LPS to the outer leaflet. Disruption of the interaction between LptD and LptE would be catastrophic to proper Lpt complex function (167). Due to the significance of LOS, immune system interference with LptD, and thus LOS transport, would severely alter gonococcal CE composition, weakening the bacterium and reducing its pathogenic capabilities.

The crystal structure of the outer membrane component of the MlaA-F system, MlaA, was recently attained from X-ray crystallography of MlaA proteins from *Serratia marcescens* and *K. pneumoniae*. MlaA is a monomeric α-helical OMP. Its six amphipathic α-helices facilitate the transport of polar phospholipid headgroups while residing within the hydrophobic interior of the outer membrane. The structure of MlaA allows diffusion of outer leaflet phospholipids exclusively (170). It is unclear how the phospholipid is transferred to MlaC, as the MlaA pore does not appear to permit the passage of acyl chains. Interactions between MlaA and MlaC have been hypothesized to induce a conformational change in MlaA, potentially by shifting helix 6 through a gate opening mechanism to enable phospholipid transfer to occur (170). *K. pneumoniae* and *S. marcescens* MlaA proteins were found in complex with the outer membrane pore OmpF in a 3:3 or a 1:3 ratio, respectively (170). The MlaA-porin interplay, mediated by van der Waals interactions, does not appear to significantly influence porin, although the presence of the porin appears to prevent MlaA aggregation (170). Normal function is based on proper architecture of the interaction and linkage of these two proteins. Targeting the maintenance of OM lipid asymmetry through a vaccine could sensitize bacteria to components of the immune system that target the outer membrane.

*Neisserial* MetQ was originally believed to bind to D-methionine (203, 204). However, structural elucidation from *Nm* revealed L-methionine in the binding pocket, which could not be displaced, even when the protein was heterologously expressed in *E. coli* in minimal medium containing only D-methionine. In support of these findings, and in contrast to the protein's annotation as a D-methionine binding protein, concrete data indicate that D-amino acids are not incorporated into proteins during ribosomal synthesis; L-amino acids are required (205). Biologically, aminoacyl-tRNA synthetases distinguish cognate L-amino acids against noncognate proteinogenic L-amino acids and also nonproteinogenic D-amino acids, thus regulating components for protein biosynthesis (206). However, *E. coli* MetQ is able to bind both D- and L-enantiomers with high affinity (207). Structural comparisons revealed similarities between *Nm* MetQ and L-methionine binding protein Tp32 from *Treponema pallidum* as well as the dipeptide GlyMet-binding protein Pg110 from *Staphylococcus aureus*, despite low sequence similarity (204). *Nm* MetQ is made up of 15 alpha helices and 10 β-strands, split into two domains, I and II, corresponding to residues 43-119 and 236-281; and 120-235, respectively. The two domains are connected by a hinge region and display a Venus flytrap-like structure, which is typical of periplasmic substrate binding proteins. The methionine binding site is within the crevasse of these two globular lobes (204). Residues critical for proper methionine coordination within the binding pocket include Arg156, which forms a salt bridge with the carboxyl group of L-methionine; Asn215, which forms one of two hydrogen bonds to the nitrogen of L-methionine; and Asn 238, which forms the second hydrogen bond and appears to be responsible for the stereospecificity of *Neisserial* MetQ (204). MetQ is a surface-displayed lipoprotein (64, 106) and thus it remains to be elucidated how L-methinonine is transported through the periplasm to the inner membrane (Figure 4).

The structure of ZnuD, a member of the TonB-dependent receptor (TbdR) family, was first crystalized in *Nm* by single isomorphous replacement, combined with opposing signals for native and seleno-derived ZnuD crystals (181). The crystal structure of ZnuD revealed a 22-stranded β-barrel pore architecture similar to that of siderophore domains common to the TbdR family, which includes the amino-terminal plug domain between residues 1-147 and its pore-forming domain from residues 148-734 on the carboxyl terminus [Figure 3E; (181)]. The plug domain of ZnuD is required for normal function of TonB in the inner membrane. In contrast to most TbdR proteins, which are involved in iron and cobalamin uptake, ZnuD was the first zinc TbdR structure solved (208). Due to the importance of metal co-factor acquisition, other gram-negative bacteria like *E.coli* use ZnuD ABC transporters to overcome zinc-deficient conditions (209). A key feature of meningococcal ZnuD is the extracellular loops. These loops are arranged in a way that displays a “comb” scaffold, allowing the uptake of zinc. Substrate binding can induce multiple conformational changes that are reversed upon zinc release (181). Key residues assisting in coordination of the zinc ion are Asp99, His100, Glu340 and His499. All four residues are highly conserved within the binding pocket across three intermediate structures (native ZnuD, ZnuD co-crystallized with cadmium, and ZnuD soaked with zinc). The binding pocket is sealed through interactions between the apical loop of the plug and extracellular loop 6. Due to the flexibility of ZnuD, remodeling of the alpha helices and β-strands occurs throughout different binding states. Zinc transport through the β-barrel channel usually requires TonB activation (181). Molecular dynamic simulations found TonB exclusively interacts with the plug domain, which is unfolded upon TonB activation (181). Further investigation using X-ray absorption spectroscopy was performed to determine whether ZnuD specifically binds zinc, or if it interacts with heme as well (181). Superimposition of the ZnuD crystal framework with the hemophore receptor HasR found a similar “lock-key” feature, although ZnuD was determined not to be a heme uptake protein (181, 210). Importantly, meningococcal ZnuD stimulates a bactericidal antibody response that recognizes peptides 233-309, 430-459, and 706-722. These peptides correspond to extracellular loops 3, 6, and 11. Loop 3 is the most immunogenic and performs a pivotal role in blocking access to the zinc binding pocket. A secondary feature of loop 3 is that it shifts to a rigid β-strand conformation upon zinc binding and back to a flexible alpha helix when the substrate is released (181). Despite the significant *Nm* ZnuD conformational changes observed upon zinc binding, its ability to elicit bactericidal antibodies suggests it may be an appropriate vaccine candidate for gonorrhea as well.

The structural studies we have discussed here not only give context to the function of our proteome-derived vaccine candidates, but also give insights into critical, surface-exposed portions of the proteins that can be targeted through structural vaccinology approaches.

## Concluding Remarks

- Proteomics-driven vaccinology for gonorrhea has begun to deliver novel antigens and determined the contents of NeMVs from four different *Ng* isolates, which can further inform the vaccine development and manufacturing processes.
- Proteomics-driven antigen discovery should be paired with comprehensive bioinformatic analyses to enable more informed decisions for rational development of subunit vaccines and facilitate the inclusion of highly conserved surface-exposed proteins with important functions.
- Our proposed approach to vaccine candidate evaluation may facilitate development of the most effective vaccine against gonorrhea in a systematic and cost-effective way suitable for an academic setting.
- The proteomics-derived antigens described participate in essential CE processes as well as pathogenesis. A subunit vaccine composed of several of these antigens has the potential to severely compromise *Ng* fitness.
- Our discovery of SliC as a previously uncharacterized virulence factor illustrates that new lessons can still be learned about *Ng* biology and also highlights the importance of considering that infection occurs in a living host and involves numerous elements that cannot be replicated *in vitro*.
- Structural vaccinology for gonorrhea is in its infancy and thus enhanced efforts should be dedicated to solving structures of all potential vaccine candidates.

## Author Contributions

FM, BB, and AS wrote the manuscript. BB and AS made final edits. AS provided illustrations.

### Conflict of Interest Statement

The authors declare that the research was conducted in the absence of any commercial or financial relationships that could be construed as a potential conflict of interest. The handling Editor declared a past co-authorship with one of the authors AS.

## Footnotes

^1^http://www.cbs.dtu.dk/services/SignalP/

^2^http://www.cbs.dtu.dk/services/LipoP/

^3^https://www.ncbi.nlm.nih.gov/COG/

^4^https://pubmlst.org/neisseria/

^5^http://www.cbs.dtu.dk/services/TMHMM/

^6^http://www.biomerieux-usa.com/clinical/etest

^7^https://biolog.com/products-portfolio-overview/phenotype-microarrays-for-microbial-cells/
